# (+)-Catechin in a 1:2 Complex with Lysine Inhibits Cancer Cell Migration and Metastatic Take in Mice

**DOI:** 10.3389/fphar.2017.00869

**Published:** 2017-12-04

**Authors:** Valéry L. Payen, Paolo E. Porporato, Pierre Danhier, Thibaut Vazeille, Marine C. N. M. Blackman, Bronislav H. May, Paul Niebes, Pierre Sonveaux

**Affiliations:** ^1^Pole of Pharmacology, Institut de Recherche Expérimentale et Clinique (IREC), Université catholique de Louvain (UCL), Brussels, Belgium; ^2^Department of Molecular Biotechnology and Health Sciences, Molecular Biotechnology Center, University of Turin, Turin, Italy; ^3^Biomedical Magnetic Resonance Research Group, Louvain Drug Research Institute, Université catholique de Louvain (UCL), Brussels, Belgium; ^4^VALORE SA, Seneffe, Belgium

**Keywords:** polyphenols, epigallocatechin gallate (EGCG), (+)-catechin, cyanidanol-3, reactive oxygen species (ROS), cancer cell migration, cancer metastasis, cancer therapy

## Abstract

Metastasis is of dismal prognosis for cancer patients, but recent evidence in mouse models of cancer shows that metastasis prevention is a reachable clinical objective. These experiments indicate that altered mitochondrial activities are associated with the metastatic phenotype. Mitochondrial transfer from metastatic to non-metastatic cells can indeed transfer the metastatic phenotype, and metastatic progenitor cells differ from other cancer cells by a higher sublethal production of mitochondrial reactive oxygen species (ROS). Moreover, mitochondria-targeted antioxidants can prevent metastatic dissemination in mouse models of cancer. Comparatively, general antioxidants have unpredictable effects on cancer metastasis, most probably because they affect several cell types, several subcellular ROS production sites and, often, several endogenous oxidant species. Thus, targeting antioxidants to mitochondria could improve their antimetastatic activities, as previously exemplified with mitochondria-targeted mitoTEMPO and mitoQ that can prevent metastatic dissemination in cancer-bearing mice. Our objective in this study was to identify whether catechins, which are known to be potent antioxidants, can inhibit cancer cell migration *in vitro* and metastatic take *in vivo*. Comparative analysis of the response to epigallocatechin-3-gallate, (+)-catechin and (+)-catechin:lysine complexes revealed that, whereas all compounds had similar general antioxidant properties, (+)-catechin:lysine 1:2, but not epigallocatechin-3-gallate, can prevent metastatic take of melanoma cells to the lungs of mice. (+)-Catechin:lysine 1:2 possesses two net positive charges provided by lysines at physiological pH, which could provide high affinity for the negatively charged mitochondrial matrix. While this study reveals that (+)-catechin:lysine 1:2 has interesting antimetastatic effects, future experiments are needed to formally demonstrate the stability of the complex, its effective tropism for mitochondria and whether or not its activity can be globally attributed to its antioxidant activity at this precise subcellular location.

## Introduction

Cancer is a progressive disease. At advanced stages, most solid tumors undergo a metastatic switch, marking the transition from local to systemic disease, which seriously limits cancer curability. Often, local interventions, such as surgery and radiotherapy, can indeed not be applied to highly metastatic tumors. Metastases are responsible for the death of ∼90% of all cancer patients (Gupta and Massague, 2006), which constitutes a strong incentive to develop and validate new treatments capable of selectively preventing or eradicating metastases.

It is estimated that tumors in the metastatic phase shed approximately one million of cancer cells per gram of tumor in the circulation every day (Liotta et al., 1974; Butler and Gullino, 1975; Chang et al., 2000). Of these cells, only few, termed metastatic progenitor cells, simultaneously possess all the characteristics needed to successfully establish a secondary tumor, including resistance to anoikis, directional migration, invasiveness, and clonogenicity. These metastatic progenitor cells represent only ∼0.01% of all circulating cancer cells (Langley and Fidler, 2011), but constitute a major target for metastatic prevention. Even if they are scarce, their characterization is possible.

Experimental work *in vitro* and in mouse models previously demonstrated that mitochondria within cancer cells control metastasis by modulating reactive oxygen species (ROS) production. The seminal work of Ishikawa et al. (2008) indeed showed that the transfer of ROS-producing mitochondria from metastatic to non-metastatic cancer cells also transfers the metastatic phenotype. More recently, based on the natural selection of superinvasive and supermetastatic cancer cells from weakly invasive and weakly metastatic precursors and their metabolic comparison, we identified mitochondrial superoxide produced at the electron transport chain (ETC) as a major metastatic trigger (Porporato et al., 2014). When produced in mitochondria, superoxide activates Src kinase directly within mitochondria, thereby activating transforming growth factor β (TGF-β) signaling independently of TGF-β. Increased superoxide production by mitochondria, whether naturally selected or experimentally induced by ETC bottlenecking (with, e.g., low dose rotenone), was sufficient to induce cancer cell migration, invasion and metastasis in mice. Tan et al. (2015) further showed that cancer cells lacking mitochondria can engulf, coopt and activate mitochondria provided by stromal cells during the metastatic cascade, thus restoring this source of ROS. Consequently, selective inactivation of mitochondrial superoxide production with mitochondria-targeted antioxidants was shown to highly significantly prevent spontaneous metastatic dissemination in mice (Porporato et al., 2014), paving the way for future clinical interventions (Porporato and Sonveaux, 2015).

General antioxidants have been the subject of intense research for cancer prevention and treatment. Because these molecules indistinctively target different cell types (including tumor-resident non-malignant host cells, some of which promote and some of which repress tumor progression), because they are not selective for a given ROS produced at a precise subcellular location and because they can interfere with anticancer therapy, general antioxidants have unpredictable effects on cancer progression (Bonner and Arbiser, 2014; Milkovic et al., 2014; Ozben, 2015; Assi and Rebillard, 2016; Russo et al., 2017). For examples, chronic vitamin E supplementation was found to significantly increase prostate cancer incidence in humans (Klein et al., 2011), vitamin C has no significant anticancer effects even when administered intravenously at high dose (Jacobs et al., 2015), and *N*-acetylcysteine can promote metastasis by improving the resistance of cancer cells to oxidative stress during cell detachment and in the blood stream (Le Gal et al., 2015). Comparatively, stronger evidence indicates that metastatic dissemination can be prevented by selectively targeting superoxide production by the dysfunctional mitochondria of metastatic progenitor cells in mice (Porporato et al., 2015), but efficient compounds must still be identified for future clinical applications.

In the present study, we aimed to determine whether catechins could be used for metastatic prevention. These polyphenols are potent antioxidants, but, even if they can modulate mitochondrial functions (Oliveira et al., 2016), they lack selectivity and can affect many signaling pathways (Singh et al., 2011). We focused on (+)-catechin (also known as cyanidanol-3), which can be extracted with high yield and purity using traditional solvent-based methods from *Uncaria gambir*, a plant found in Southeast Asia (Niebes et al., 1981). In theory, this compound is more stable than epigallocatechin-3-gallate (EGCG, the main catechin found in green tea and currently tested in 37 clinical trials for cancer patients ^1^), which undergoes auto-oxidation and epimerization in water (Das and Griffiths, 1967; Sang et al., 2005). Of further interest, (+)-catechin has been reported to form complexes with one or two lysines, which further increases its water solubility and brings a net positive charge (-NH_3_^+^) to the catechin at physiological pH (Niebes et al., 1981). The net positive charge of these complexes is of interest, because it could target (+)-catechin to the negatively charged mitochondrial matrix, in a way similar to antioxidants 2,2,6,6-tetramethylpiperidine 1-oxyl (TEMPO) and coenzyme Q10 that can be targeted to mitochondria when a positively charged triphenylphosphonium (TPP) group is chemically grafted on these molecules to yield mitoTEMPO and mitoQ, respectively (Murphy, 2008). Using EGCG as a reference compound, we therefore studied the potential antimetastatic effects of (+)-catechin and (+)-catechin:lysine complexes *in vitro* and *in vivo*.

## Materials and Methods

### Cells and Cell Culture

Wild-type B16F10 mouse melanoma and SiHa human cervix adenocarcinoma cells were from ATCC. Superinvasive SiHa-F3 cells were obtained by serial *in vitro* selection, as previously described (Porporato et al., 2014). Cell lines were routinely cultured at subconfluence for no more than 15 passages in DMEM containing 4.5 g/L glucose and GlutaMAX (Gibco), supplemented with 10% FBS (Sigma–Aldrich) and 1% penicillin/streptomycin (Sigma–Aldrich) in a humidified atmosphere with 5% CO_2_ at 37°C.

### Chemicals

(+)-Catechin [(2*R*,3*S*)-2-(3,4-dihydroxyphenyl)-3,4-dihydro-1(2H)-benzopyran-3,5,7-triol], also known as cyanidanol-3, was extracted and purified from *U. gambir* according to Niebes et al. (1981), reaching ≥99% purity. EGCG and *D/L*-lysine monohydrochloride were from Sigma–Aldrich. For the production of catechin/lysine complexes, catechin powder was mixed with *DL*-lysine monohydrochloride at a 1:1 or 1:2 (mol/mol) ratio before dissolution in culture medium buffered at pH 7.4. Where indicated, rotenone was used at low nanomolar concentration in order to promote mitochondrial superoxide production, cell migration and experimental metastasis, according to Porporato et al. (2014). Unless specified otherwise, all other chemicals were from Sigma–Aldrich.

### Cell Viability Assay

Twenty thousand SiHa-F3 or B16F10 cells per well were seeded in a 96-well plate containing culture medium together with test compounds, and incubated for 16 h. Cells were washed with PBS, fixed in methanol for 3 min, stained with a 0.23% water solution of Crystal Violet for 30 min, and washed with water. Crystal Violet precipitates were resuspended in 200 μl of DMSO, and absorbance was measured at 595 nm with a Victor X4 spectrophotometer (Perkin-Elmer).

### MTT Assay

Twenty thousand SiHa-F3 cells per well were seeded in a 96-well plate containing culture medium together with test compounds, and incubated for 16 h. Cells were washed with HBSS buffer (containing Ca^2+^/Mg^2+^ and 10 mM of HEPES), and incubated for 3 h with a saturated solution of 3-(4,5-dimethylthiazol-2-yl)-2,5-diphenyltetrazolium bromide (MTT, Calbiochem) in HBSS. Formazan precipitates were resuspended in 50 μl of DMSO, and absorbance was measured at 570 nm with a Victor X4 spectrophotometer.

### ROS Measurements

Twenty thousand SiHa-F3 or B16F10 cells per well were seeded in a 96-well plate containing culture medium. On the next day, they were treated with rotenone and/or test compounds or vehicle for 1 h (SiHa-F3) or 6 h (B16F10), washed with HBSS, and ROS were measured using 1 μM of CM-H_2_-dichlorofluorescein-diacetate (CM-H_2_-DCFDA, Life technologies) as previously reported (Porporato et al., 2014). Fluorescence emission at 535 nm following excitation at 490 nm was measured with a Victor X4 spectrophotometer, and data were normalized to protein content (Bradford).

### Cell Migration

A 48-well micro-chemotaxis chamber (8-μm diameter pores; Neuroprobe) and transwell inserts (Corning #353097) were used as previously described (Porporato et al., 2014), with 0.15% (SiHa-F3) or 0.5% (B16F10) FBS as a chemo-attractant. Fifty thousand SiHa-F3 cells or two hundred thousand B16F10 cells per well were allowed to migrate during 16 h. Migrated cells were then fixed with methanol, stained with Crystal Violet, and imaged with an inverted phase Axiovert microscope equipped with a Mrc camera (Zeiss). Cells were counted using the ImageJ software (NIH). Data were normalized to cell viability.

### *In Vivo* Metastasis Assays

All *in vivo* experiments were conducted under approval of the Université catholique de Louvain (UCL) authorities (*Comité d’Ethique Facultaire pour l’Expérimentation Animale*) according to national animal care regulations. Specific authorizations were UCL/2012/MD/005 and UCL/2016/MD/018. Experimental metastasis was conducted to assess metastatic take as in Porporato et al. (2014). Briefly, B16F10 melanoma cells were treated for 6 h with test compound, rotenone (20 nM dissolved in 0.2% DMSO to induce mitochondrial superoxide production) or vehicle (DMSO). One million cells were then injected in the tail vein of 8-weeks-old syngeneic C57BL/6JRj male mice (Janvier). Mice were sacrificed 2 weeks later. Their lungs were insufflated with a saline solution, and melanin-containing lung metastases were counted under a Stemi 2000-C dissection microscope (Zeiss) equipped with a Mrc camera.

### Statistics

Data show means ± SEM. *N* refers to the number of independent experiments and *n* to the total number of replicates per treatment condition. Student’s *t*-test, one-way ANOVA with Dunnett or Bonferroni *post hoc* tests and two-way ANOVA on unmatched values with Dunnett *post hoc* test were used as indicated. *P* < 0.05 was considered to be statistically significant. In some graphs, SEM are smaller than symbols.

## Results

### Epigallocatechin-3-gallate Is Significantly More Antioxidant in SiHa-F3 Cancer Cells than (+)-Catechin, (+)-Catechin:Lysine 1:1 and (+)-Catechin:Lysine 1:2

**FIGURE 1:**
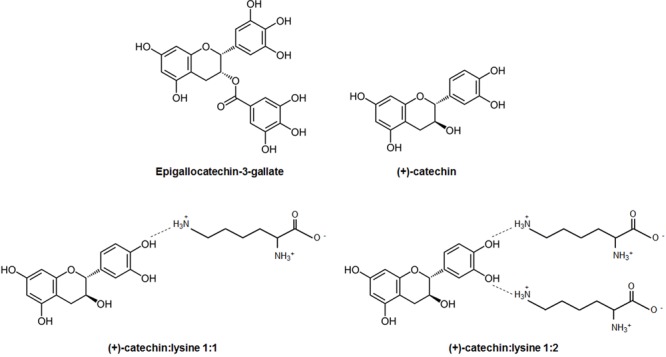
Chemical structures. Shown are the chemical structures of the catechins used in this study, as well as models of their interactions with lysines to form complexes.

This study aimed to investigate the antimetastatic potential of catechins. EGCG served as a reference compound, and its antimetastatic potential was compared to that of (+)-catechin complexed or not with one or two molecules of lysine (**Figure 1**). Compared to EGCG and (+)-catechin that are general antioxidants, lysine in complexes with catechins could provide a positive charge for mitochondrial targeting. For *in vitro* assays, our model cell line was SiHa-F3, a superinvasive derivative of human SiHa cervix adenocarcinoma cells that was produced by *in vitro* selection and thoroughly characterized in a previous study (Porporato et al., 2014). Compared to wild-type SiHa, SiHa-F3 cells produce more mitochondrial superoxide, which directly accounts for their increased migratory activity, as previously demonstrated with selective mitochondrial superoxide inhibitor mitoTEMPO (Porporato et al., 2014).

**FIGURE 2:**
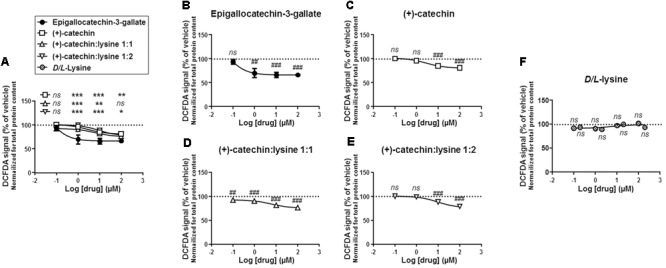
(+)-Catechin derivatives and epigallocatechin-3-gallate (EGCG) have similar general antioxidant effects in SiHa-F3 cancer cells. **(A–E)** Reactive oxygen species (ROS) level in SiHa-F3 cells was measured after a 1 h treatment with increasing doses of EGCG (*N* = 2, *n* = 8–16), (+)-catechin (*N* = 2, *n* = 16), (+)-catechin in a 1:1 complex with lysine (*N* = 3, *n* = 24–32), and (+)-catechin in a 1:2 complex with lysine (*N* = 2, *n* = 16–24). The five panels show the same sets of data with different statistical analyses to compare individual treatments to reference compound EGCG **(A)** or to vehicle treatment set as 100% **(B–E)**. **(A)** The graph shows the dose-response curves of the four treatments for comparison. **(B)** Dose-response curve for EGCG. **(C)** Dose-response curve for (+)-catechin. **(D)** Dose-response curve for (+)-catechin:lysine 1:1. **(E)** Dose-response curve for (+)-catechin:lysine 1:2. **(F)** ROS level in SiHa-F3 cells treated for 1 h with increasing doses of *D/L*-lysine monohydrochloride (*N* = 2, *n* = 16). All data are shown as means ± SEM. ^∗^*P* < 0.05, ^∗∗^*P* < 0.01, ^∗∗∗^*P* < 0.005 compared to a same dose of EGCG using two-way ANOVA; ^##^*P* < 0.01, ^###^*P* < 0.005 compared to vehicle treatment using one-way ANOVA with Dunnett’s multiple comparison test.

We first compared the different compounds for their antioxidant activities in SiHa-F3 cells. Cells were exposed to increasing doses of catechins (100 nM to 100 μM) during 1 h, after which total ROS levels were measured using fluorescent reporter CM-H_2_-DCFDA. Side-by-side comparison revealed that EGCG was significantly more antioxidant than (+)-catechin, (+)-catechin:lysine 1:1 and (+)-catechin:lysine 1:2 at all the doses that we tested, except at 100 μM where catechin:lysine 1:1 had a similar antioxidant activity compared to EGCG (**Figure 2A**). Comparison to vehicle treatment was used to identify the lowest dose at which each compound exerted a significant antioxidant effect. This dose corresponded to 1 μM for EGCG (**Figure 2B**), 10 μM for (+)-catechin (**Figure 2C**), 100 nM for (+)-catechin:lysine 1:1 (**Figure 2D**), and 10 μM for (+)-catechin:lysine 1:2 (**Figure 2E**). Of note, *D*/*L*-lysine alone did not affect the total ROS levels of SiHa-F3 cells at concentrations ranging from 100 nM to 200 μM (**Figure 2F**).

### Epigallocatechin-3-gallate Is Significantly More Cytotoxic for SiHa-F3 Cancer Cells than (+)-Catechin, (+)-Catechin:Lysine 1:1 and (+)-Catechin:Lysine 1:2

**FIGURE 3:**
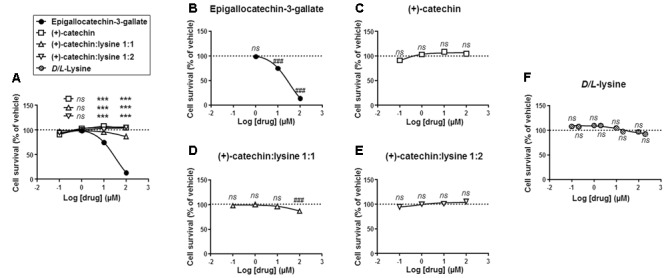
(+)-Catechin and its complexes with lysine are less cytotoxic for SiHa-F3 cancer cells than EGCG. **(A–E)** SiHa-F3 cell viability was measured by crystal violet staining following a 16 h treatment with increasing doses of EGCG (*N* = 2, *n* = 24), (+)-catechin (*N* = 2, *n* = 16–24), (+)-catechin in a 1:1 complex with lysine (*N* = 2, *n* = 15–16), and (+)-catechin in a 1:2 complex with lysine (*N* = 2, *n* = 16–24). The five panels show the same sets of data with different statistical analyses to compare individual treatments to reference compound EGCG **(A)** or to vehicle treatment set as 100% **(B–E)**. **(A)** The graph shows the dose-response curves of the four treatments for comparison. **(B)** Dose-response curve for EGCG. **(C)** Dose-response curve for (+)-catechin. **(D)** Dose-response curve for (+)-catechin:lysine 1:1. **(E)** Dose-response curve for (+)-catechin:lysine 1:2. **(F)** SiHa-F3 cell viability was measured by crystal violet staining following a 16 h treatment with increasing doses of *D/L*-lysine monohydrochloride (*N* = 2, *n* = 15–16). All data are shown as means ± SEM. ^∗∗∗^*P* < 0.005 compared to a same dose of EGCG using two-way ANOVA; ^###^*P* < 0.005 compared to vehicle treatment using one-way ANOVA with Dunnett’s multiple comparison test.

We next tested the cytotoxic activity of the different catechins. SiHa-F3 cells were exposed to increasing doses of catechins for 16 h, after which their survival was determined using crystal violet staining. Side-by-side comparison showed that EGCG was significantly more toxic to SiHa-F3 cells than the other catechins at doses ≥10 μM (**Figure 3A**). The lowest dose inducing cytotoxicity was 10 μM for EGCG (**Figure 3B**). (+)-Catechin did not kill SiHa-F3 cancer cells up to 100 μM (**Figure 3C**), (+)-catechin:lysine 1:1 induced significant yet limited cytotoxicity at a dose of 100 μM (**Figure 3D**), and (+)-catechin:lysine 1:2 was not toxic at all the tested doses (**Figure 3E**). *D*/*L*-lysine alone did not affect SiHa-F3 cell viability (**Figure 3F**).

**FIGURE 4:**
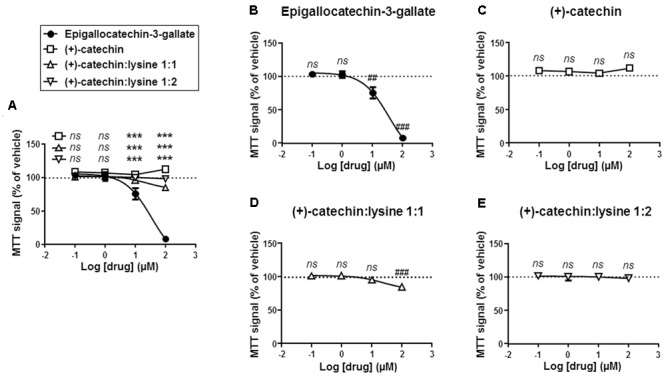
(+)-Catechin and its complexes with lysine are less metabolically toxic for SiHa-F3 cancer cells than EGCG. **(A–E)**, The activity of NAD(P)H-dependent oxidoreductases in SiHa-F3 cells was measured with a MTT assay following a 16-h treatment with increasing doses of EGCG (*N* = 2, *n* = 12), (+)-catechin (*N* = 2, *n* = 12), (+)-catechin in a 1:1 complex with lysine (*N* = 2, *n* = 16), and (+)-catechin in a 1:2 complex with lysine (*N* = 2, *n* = 12). The five panels show the same sets of data with different statistical analyses to compare individual treatments to reference compound EGCG **(A)** or to vehicle treatment set as 100% **(B–E)**. **(A)** The graph shows the dose-response curves of the four treatments for comparison. **(B)** Dose-response curve for EGCG. **(C)** Dose-response curve for (+)-catechin. **(D)** Dose-response curve for (+)-catechin:lysine 1:1. **(E)** Dose-response curve for (+)-catechin:lysine 1:2. All data are shown as means ± SEM. ^∗∗∗^*P* < 0.005 compared to a same dose of EGCG using two-way ANOVA; ^##^*P* < 0.01, ^###^*P* < 0.005 compared to vehicle treatment using one-way ANOVA with Dunnett’s multiple comparison test.

Comparison was extended to cell metabolism, and we used a MTT assay reporting on the activity of intracellular NAD(P)H-dependent oxidoreductases (Berridge and Tan, 1993). In this assay, MTT reduction reflects the status of the NAD(P)H pool inside cells. Side-by-side comparison showed that EGCG reduced MTT oxidation more significantly than the other compounds at a dose ≥10 μM (**Figure 4A**). The lowest effective dose was 10 μM for EGCG (**Figure 4B**). (+)-catechin did not modify MTT reduction (**Figure 4C**), (+)-catechin:lysine 1:1 had a slight but significant effect at 100 μM (**Figure 4D**), and (+)-catechin:lysine 1:2 did not modify MTT reduction (**Figure 4E**). These data closely matched those obtained in the cytotoxic assay with crystal violet.

### (+)-Catechin:Lysine 1:1 and (+)-Catechin:Lysine 1:2 Inhibit Cancer Cell Migration Independently of Cancer Cell Killing

Cell migration is a fundamental characteristic of metastatic progenitor cells (Gupta and Massague, 2006), and SiHa-F3 cells were previously shown to be highly migratory compared to wild-type SiHa (Porporato et al., 2014). Their increased migratory activity has been demonstrated to be directly dependent on an elevated production of mitochondrial superoxide (Porporato et al., 2014). Because the matrix of functional mitochondria is negatively charged due to the proton-motive force and since, unlike EGCG, catechin:lysine complexes have a net positive charge (-NH_3_^+^ in lysine), we hypothesized that catechin:lysine complexes could inhibit SiHa-F3 cell migration more efficiently than EGCG and (+)-catechin.

**FIGURE 5:**
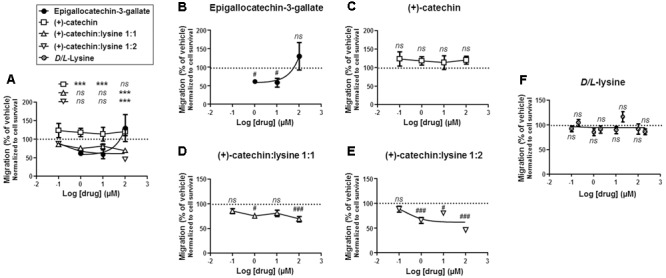
(+)-Catechin:lysine complexes and EGCG, but not (+)-catechin, inhibit SiHa-F3 cancer cell migration. **(A–E)** SiHa-F3 cancer cell migration was evaluated with a Boyden chamber assay using FBS (0.15%) as chemoattractant and 16 h as an endpoint. Cells were treated with increasing doses of EGCG (*N* = 2, *n* = 6–18), (+)-catechin (*N* = 2, *n* = 6–12), (+)-catechin in a 1:1 complex with lysine (*N* = 2, *n* = 11–12), and (+)-catechin in a 1:2 complex with lysine (*N* = 3, *n* = 17–18). The five panels show the same sets of data with different statistical analyses to compare individual treatments to reference compound EGCG **(A)** or to vehicle treatment set as 100% **(B–E)**. **(A)** The graph shows the dose-response curves of the four treatments for comparison. **(B)** Dose-response curve for EGCG. **(C)** Dose-response curve for (+)-catechin. **(D)** Dose-response curve for (+)-catechin:lysine 1:1. **(E)** Dose-response curve for (+)-catechin:lysine 1:2. **(F)** SiHa-F3 cancer cell migration was evaluated with a Boyden chamber assay using FBS (0.15%) as chemoattractant and 16 h as an endpoint. Cells were treated with increasing doses of *D/L*-lysine monohydrochloride (*N* = 2, *n* = 12). All data are shown as means ± SEM. ^∗∗∗^*P* < 0.005 compared to a same dose of EGCG using two-way ANOVA; ^#^*P* < 0.05, ^###^*P* < 0.005 compared to vehicle treatment using one-way ANOVA with Dunnett’s multiple comparison test.

The anti-migratory potential of increasing doses of catechins was tested in a micro-chemotaxis chamber with 0.15% of FBS as chemo-attractant, and the cells were allowed to migrate during 16 h. Results were normalized to cell death induced by the different treatments (**Figure 3A**). Side-by-side comparison showed that surviving SiHa-F3 cells treated with (+)-catechin:lysine 1:1 and (+)-catechin:lysine 1:2 were significantly less migratory compared to cells treated with EGCG at a dose of 100 μM (**Figure 5A**). Compared to vehicle, EGCG was inefficient to prevent cancer cell migration independently of cancer cell killing at a dose of 100 μM (**Figure 5B**). (+)-Catechin did not influence the migratory activity of the cells (**Figure 5C**). Comparatively, (+)-catechin:lysine 1:1 (**Figure 5D**) and (+)-catechin:lysine 1:2 (**Figure 5E**) significantly decreased cancer cell migration. (+)-Catechin:lysine 1:2 was the most effective, with a reduction in cancer cell migration by ∼35 to ∼50% at doses ≥1 μM. (+)-Catechin:lysine 1:1 inhibited ∼25–30% cancer cell migration at doses of 1 and 100 μM, with no statistically significant effects at 10 μM (*P* = 0.06). *D*/*L*-lysine alone did not affect SiHa-F3 cell migration (**Figure 5F**).

A comparative analysis of the effects of catechins on SiHa-F3 cells is shown in **Table 1**.

**Table 1:** Activities of catechin derivatives on SiHa-F3 cancer cells.

	EGCG	(+)-Catechin	(+)-Catechin:lysine 1:1	(+)-Catechin:lysine 1:2
Concentration for ROS inhibition^∗^	≥1 μM	≥10 μM	≥0.1 μM	≥10 μM
Cytotoxic concentration^#^	≥10 μM	>100 μM	≥100 μM	>100 μM
Concentration for NAD(P)H oxidase inhibition^$^	≥10 μM	>100 μM	≥100 μM	>100 μM
Antimigratory concentrationˆ	≥1 μM and <100 μM independently of cytotoxicity	>100 μM	≥1 μM	≥1 μM

*Determined with: ^∗^CM-H_2_-DCFDA, ^#^crystal violet, ^$^MTT, ˆtranswell assays with 0.15% FBS as chemoattractant.*

### (+)-Catechin:Lysine 1:2, But Not Epigallocatechin-3-gallate, Inhibits Experimental Melanoma Metastasis in Mice

**FIGURE 6:**
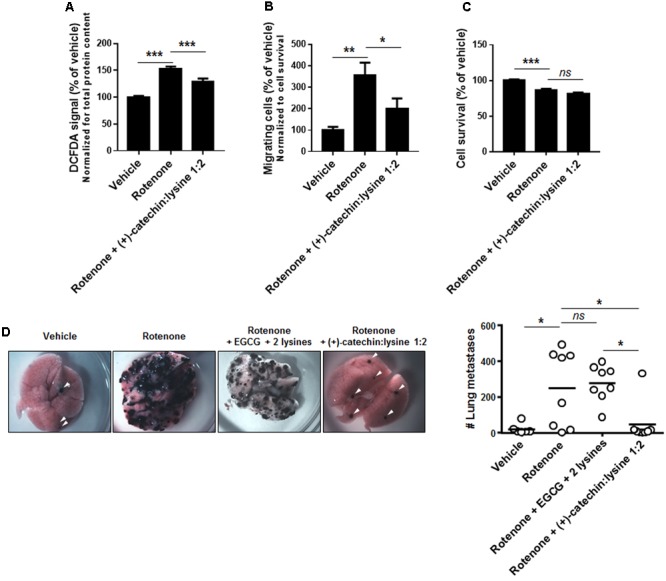
(+)-Catechin in a 1:2 complex with lysine, but not EGCG, inhibits the metastatic take of B16F10 melanoma cell in the lungs of mice. **(A–C)** B16F10 mouse melanoma cells were treated with vehicle (DMSO 0.1%) and rotenone (20 nM) alone or in combination with 10 μM of (+)-catechin in a 1:2 complex with lysine. **(A)** ROS level in cancer cells was measured after a 6 h treatment (*N* = 2, *n* = 16). **(B)** Cancer cell migration was evaluated with a Boyden chamber assay using FBS (0.5%) as chemoattractant and 16 h as an endpoint (*N* = 2, *n* = 8). **(C)** Cancer cell viability was measured by crystal violet staining following a 16 h treatment (*N* = 2, *n* = 16). **(D)** Metastatic take to the lungs was determined in C57BL/6JRj mice that received a tail vein injection of 1,000,000 syngeneic B16F10 melanoma cells. Cancer cells were pretreated for 6 h with vehicle (DMSO 0.2%; *n* = 6) or rotenone alone (20 nM; *n* = 8) or in combination with EGCG + 2 lysines mol/mol (10 μM; *n* = 8) or in combination with (+)-catechin in a 1:2 complex with lysine (10 μM; *n* = 8). Representative pictures of the lungs are shown, and the graph displays the number of metastasis in both lungs for each animal. Dots represent individual mice and the bars the group average. ^∗^*P* < 0.05, ^∗∗^*P* < 0.01, ^∗∗∗^*P* < 0.005, ns: *P* > 0.05 using one-way ANOVA with Dunnet’s **(A–C)** or Bonferroni’s **(D)** multiple comparison test.

Finally, we evaluated the capability of catechins to decrease metastatic take in mice *in vivo*. SiHa cells are only weakly metastatic in SCID mice (Cairns and Hill, 2004), and SiHa-F3 cells did not generate quantifiable lung metastases (*data not shown*). We therefore used spontaneously metastatic B16F10 cancer cells that were pretreated with catechins and administered intravenously to immunocompetent syngeneic C57BL/6JRj mice. B16F10 cells are weakly metastatic, but the proportion of metastatic progenitor cells, hence metastatic take in the lungs after tail vein injection, can be increased by triggering mitochondrial superoxide production with a nanomolar dose of rotenone (Porporato et al., 2014).

(+)-Catechin:lysine 1:2 had shown to be the most potent among the tested compounds to inhibit SiHa-F3 cell migration (**Figure 5**). We therefore tested the effects of the complex on rotenone-treated B16F10 cells. In agreement with the results obtained with SiHa-F3, 10 μM of (+)-catechin:lysine 1:2 reduced ROS production (**Figure 6A**) and the migration of B16F10 cancer cells (**Figure 6B**) without affecting cell survival (**Figure 6C**). Rotenone, used at a low dose to promote ROS production by the ETC (**Figure 6A**) and cell migration (**Figure 6B**; Porporato et al., 2014), had a limited yet significant cytotoxic effect after 16 h of treatment (**Figure 6C**). Together, this set of data validated our model for next *in vivo* assays.

For *in vivo* experiments, B16F10 cells were treated for 6 h with 20 nM of rotenone (to render the cells highly metastatic) ± 10 μM of EGCG + 2 lysines (mol/mol) or (+)-catechin:lysine 1:2 or vehicle (DMSO 0.2%) before intravenous injection. Data revealed that (+)-catechin:lysine 1:2, but not EGCG + 2 lysines, significantly prevented rotenone-induced metastatic take in the lungs of mice (**Figure 6D**).

## Discussion

This study aimed to test whether catechins have the capability to prevent tumor metastasis. We report that, among tested compounds, (+)-catechin:lysine 1:2 prevents tumor take of mouse melanoma cells in mouse lungs, unveiling a particularly attractive compound for future developments ultimately aimed to repress metastatic cancer dissemination.

Mitochondrial superoxide promotes cancer metastasis by increasing mitochondrial H_2_O_2_ levels and activating Src kinase, downstream intermediates of the TGF-β pathway and focal adhesion kinase family member Pyk2 (Porporato et al., 2014), the latter acting as a prometastatic effector (Wendt et al., 2013). Experimentally, increased mitochondrial superoxide production can result from a natural selection of metastatic progenitor cell traits or from increased electron leak when the ETC is partially inhibited with, e.g., a low dose of rotenone. Such models, namely SiHa-F3 and rotenone-treated B16F10 cancer cells (Porporato et al., 2014), were used in the present study. Because mitochondrial ROS promote tumor metastasis, mitochondrial ROS scavenging interferes with the metastatic cascade. Indeed, it was previously demonstrated that metastatic prevention can be achieved in mice with mitochondria-targeted superoxide scavenger mitoTEMPO (Dikalova et al., 2010; Nazarewicz et al., 2013; Porporato et al., 2014). In this study, our aim was to identify other compounds for future clinical applications.

We focused on catechins for their known antioxidant activities, and we further reasoned that positively charged (+)-catechin:lysine complexes could be of particular interest for their theoretical mitochondrial tropism. Accordingly, our *in vitro* data evidenced that (+)-catechin:lysine complexes have different effects than (+)-catechin. Although all tested (+)-catechins had similar antioxidant properties (comparable dose-response curves), only (+)-catechin:lysine 1:1 and (+)-catechin:lysine 1:2 were capable of inhibiting cancer cell migration at concentrations ≤100 μM. (+)-Catechin did not inhibit cancer cell migration at all, and EGCG, the most potent antioxidant that we tested, did not impair the migration of surviving cells. Thus, the antimigratory activities of catechins are not linked to their general antioxidant potential. Our *in vivo* data further showed that (+)-catechin:lysine 1:2, which possesses two positive charges at physiological pH, is more active at inhibiting metastatic take compared to EGCG + 2 lysines. One possibility to explain this observation is that (+)-catechin:lysine complexes, preferentially (+)-catechin:lysine 1:2, inhibit mitochondrial ROS generation with some selectivity in metastatic progenitor cells. However, mitochondria-targeted superoxide probe MitoSOX (Roelofs et al., 2015) was not sensitive enough to measure changes of mitochondrial ROS production under basal conditions in our model. Another possibility is that (+)-catechin:lysine 1:2 inhibits metastatic take independently of its antioxidant activity. However, the hypothesis of a mitochondria-selective antioxidant action of (+)-catechin:lysine 1:2 in the prevention of metastatic take is more likely, based on the fact that metastatic take in our model primarily depends on increase mitochondrial ROS production by low nanomolar doses of rotenone (Porporato et al., 2014). It is of course evident that future studies are needed to experimentally demonstrate that (+)-catechin:lysine 1:2 accumulates in mitochondria where it acts as an antioxidant, with as secondary aim to verify transmembrane trafficking of this relatively highly hydrophilic compound compared to more lipophilic mitoTEMPO. As an alternative or in addition to enhanced mitochondrial tropism, (+)-catechin:lysine 1:2 could have more selectivity for prometastatic pathways compared to other polyphenols. Reference compound EGCG can indeed modulate a large array of signaling pathways and enzymes, including JAK/STAT, MAPK, PI_3_K/Akt, Wnt, Notch, DNA methyl-transferases, dihydrofolate reductase, and glutamate dehydrogenase, to name only a few (Li C. et al., 2006; Singh et al., 2011). Pleiotropic effects of EGCG can further explain its propensity to decrease NAD(P)H oxidase activity (Li H. L. et al., 2006 and our MTT assays) and to kill cancer cells (Kil et al., 2011; Yu et al., 2014). Paradoxically, EGCG can have pro-oxidant activities: upon oxidation, it forms auto-oxidative quinone derivatives that generate superoxide and the hydroxyl radical (Azam et al., 2004).

An important question raised by our study is the stable formation of catechin:lysine complexes. Although direct chemical evidence is currently lacking, we provide biological evidence in favor of the existence of such complexes, at least in the case of (+)-catechin. First, different responses of cancer cells to (+)-catechin, (+)-catechin:lysine 1:1 and (+)-catechin:lysine 1:2 revealed that the presence of lysine is important. However, adding lysine was not sufficient to provide antimetastatic properties, as illustrated by the inability of EGCG + 2 lysines to interfere with metastatic take *in vivo*, whereas (+)-catechin:lysine 1:2 was highly effective. Lysine alone had no effects. Second, 1-mM stock solutions of the complexes were made before experiments, and all *in vitro* experiments were conducted in commercial DMEM that contains 800 μM of *L*-lysine. The fact that (+)-catechin in these conditions did not inhibit cancer cell migration, whereas (+)-catechin:lysine 1:1 and (+)-catechin:lysine 1:2 did at doses ≥1 μM, suggests that stable complexes were formed in stock solutions but not in DMEM. (+)-Catechin:lysine 1:1 and (+)-catechin:lysine 1:2 are therefore likely to be relatively stable chemical entities governed by hydrogen bounds and charge interactions, as proposed in **Figure 1**. Finally, EGCG admixed with 2 lysines had no antimetastatic effects at the concentrations that we tested, even if EGCG was at least as antioxidant as (+)-catechin:lysine 1:2. It is therefore possible that, in contrast with (+)-catechin characterized by a diphenol group, EGCG with two triphenol groups does not form stable complexes with lysines. This could possibly be due to steric hindrance and charge repulsion when lysines interact with a triphenol, a hypothesis that requires chemical evaluation. Alternatively, if EGCG:lysine complexes would be formed, they may not reach mitochondria.

Of note, EGCG and (+)-catechin could have antimetastatic effect in models of spontaneous metastasis, where EGCG can interfere with pathways controlling early steps of the metastatic process (Khan and Mukhtar, 2010; Maruyama et al., 2014; Zhang et al., 2016) and (+)-catechin with the degradation of collagen (Menon et al., 1999). Molecularly, (+)-catechin was reported to increase the number of pepsin-resistant cross-links in collagen, rendering collagen less soluble and more resistant to collagenase than native collagen (Niebes, 1977; Pontz et al., 1982). However, the effect of (+)-catechin on collagen does not explain decreased metastatic take in our model, as collagen of recipient mice was never exposed to (+)-catechin. Of further note, our data also show that high doses of EGCG do not inhibit cancer cell migration independently of cancer cell killing. This could be linked to the pleiotropic actions of EGCG, but this hypothesis, at this stage of research, is still largely speculative. More generally, we do not believe that general antioxidants are reliable to treat cancer because they interfere non-selectively with many redox systems in malignant as well as in non-malignant cells.

## Conclusion

We report that (+)-catechin:lysine 1:2 significantly inhibits cancer cell migration in a model of superinvasive human cervix cancer cells *in vitro* and highly significantly inhibits metastatic take in a mouse melanoma model *in vivo*. This property was not shared with (+)-catechin or EGCG. Even if not definitive, we further provide elements suggesting that (+)-catechin:lysine 1:2 is a stable complex. While further experiments are needed to confirm the antimetastatic effects of (+)-catechin:lysine 1:2 in additional cancer models including in mice bearing primary metastatic tumors and treated systemically (this will require to determine the stability of the complex *in vivo*), we believe that our study brings concrete elements in support of the development of catechin:lysine complexes for metastatic prevention in cancer.

## Author Contributions

VP led the experimental work. VP, PP, PD, TV, and MB performed the experiments. VP, PP, PD, TV, MB, and PS analyzed the data. BM, PN, and PS designed the experimental approach. BM and PN provided test compounds. VP and PS wrote the manuscript. PS obtained financial support and directed the study. VP, PP, PD, TV, MB, BM, PN, and PS critically reviewed the manuscript.

## Conflict of Interest Statement

Manuscript data are included in patent application WO2016/075161A1, of which some of the authors are inventors. VALORE SA owns patent application WO2014/184197A1 on the use of (+)-catechin:lysine complexes. Ongoing follow-up studies are financed in part by BePharBel Manufacturing and the Région Wallonne de Belgique (projects CATECHIN-1 and CATECHIN-2). The other authors declare that the research was conducted in the absence of any commercial or financial relationships that could be construed as a potential conflict of interest.

## Footnotes

1 clinicaltrials.gov
